# Molecular identification of *Burkholderia cepacia* complex from people with cystic fibrosis in northwestern Iran: a research note

**DOI:** 10.1186/s13104-026-07838-y

**Published:** 2026-05-16

**Authors:** Amir Hossein Jafari-Rouhi, Hassan Tizfahm Tikmehdash, Nader Mosavari, Leila Vahedi

**Affiliations:** 1 Tuberculosis and Lung Diseases Research Center, Tabriz University of Medical Sciences, Tabriz, Iran; 2 Scientific-Applied Education Center of Ostad Shahryar, Tabriz, Iran; 3 Reference Laboratory of Bovine Tuberculosis, Razi Vaccine and Serum Research Institute, Agricultural Research Education and Extension Organization (AREEO), Karaj, Iran; 4 Road Traffic Injury Research Center, Tabriz University of Medical Sciences, Tabriz, Iran

**Keywords:** *Burkholderia cepacia* complex, Cystic Fibrosis, Clinical presentation, PCR, ELISA

## Abstract

**Objective:**

The objective of this study was to identify the *Burkholderia cepacia* complex in patients with cystic fibrosis in northwestern Iran using conventional culture. Additionally, the study aimed to evaluate the efficacy of molecular identification via PCR and a preliminary ELISA, and to determine the correlation between laboratory findings and clinical presentations.

**Results:**

The *Burkholderia cepacia* complex was not identified in oral mucosal swab samples from 100 cystic fibrosis patients using conventional culture media, including MacConkey agar and blood agar, followed by biochemical tests. However, the complex was identified with high precision by targeting the *recA* gene locus in PCR analysis. Furthermore, an optimized indirect ELISA using native antigens identified five and 12 positive cases, respectively. In the five PCR-positive cases, symptoms included growth delay, productive cough, and digital clubbing, accompanied by radiological findings such as bronchiolar involvement, airway wall thickening, and bronchiectasis. All five cases responded positively to co-trimoxazole treatment. These findings suggest that molecular analyses and ELISA assays using native species could be promising candidates for establishing efficient and appropriate methods for the diagnosis of the *Burkholderia cepacia* complex.

## Introduction

Cystic fibrosis (CF) is a recessive disorder characterized by respiratory tract infections, gastrointestinal abnormalities and nutritional dysfunction, sweat gland malfunction, and male infertility [1, 2]. Lung complications are the primary cause of morbidity and mortality in individuals with CF [3].

Among opportunistic pathogens, the *Burkholderia cepacia* complex (*BCC*)—comprising at least 22 species — poses a major threat associated with severe pulmonary infections, accelerated decline in lung function, and reduced survival in CF populations. Person-to-person transmission and high intrinsic resistance to multiple antibiotics complicate treatment strategies [4, 5].

In low-income countries, the diagnosis of *BCC* is primarily conducted using conventional culture on non-selective media and phenotypic tests. However, these methods are time-consuming and prone to technical issues [6, 7]. Such limitations have been shown to increase the risk of misdiagnosis, the administration of inappropriate therapy, and nosocomial transmission [8, 9].

In Iran, available data on the prevalence of *BCC* in people with CF are limited. A 2004 study on 53 CF respiratory samples reported a BCC prevalence of approximately 11%. Furthermore, a 2014 study involving 100 CF populations at Masih Daneshvari Hospital reported a prevalence of 5%, emphasizing the role of molecular methods in enhancing diagnostic accuracy [10, 11].

The high burden of infectious diseases in low-resource settings highlights the limitations of traditional diagnostic methods regarding diagnosis and treatment. Recent research indicates that molecular testing, particularly PCR, provides markedly greater sensitivity and specificity than culture-based methods in these settings. In low-income countries, barriers such as insufficient or improper equipment and a scarcity of trained staff hinder the prompt and precise identification of pathogens. These issues increase the likelihood of misdiagnosis or overlooked infections [9, 12]. Therefore, given the presence of *BCC* in the Iranian CF population and its serious threat, it is essential to evaluate sensitive diagnostic approaches for the timely identification of this pathogen. This underscores the importance of conducting the present study in northwestern Iran.

## Main text

### Methods

#### Participants and samples

The current study was conducted on patients with CF who were admitted to or followed up at the Children’s Educational and Training Hospital of Tabriz University of Medical Sciences, the largest governmental and referral hospital in northwestern Iran, from September 22, 2023, to June 21, 2024. All participants were registered in the Iranian National Cystic Fibrosis Registry, which was established by the Ministry of Health and Medical Education in 2017 [3, 13].

The diagnosis of CF was made using the Cystic Fibrosis Foundation Consensus Guidelines [14]. The diagnosis was based on the presence of specific symptoms, such as respiratory complications, gastrointestinal abnormalities, salt-loss syndromes, and genital abnormalities, together with either an elevated sweat chloride concentration (> 60 mEq/L) measured by the Gibson and Cooke method or the identification of disease-causing mutations in the *CFTR* (cystic fibrosis transmembrane conductance regulator) gene [15].

For microbiological analysis, the swabs were collected from the oral mucosa of 100 patients with CF. Mucosal swabs were utilized instead of sputum samples because the patients were young children and invasive methods posed ethical challenges; therefore, this sampling method was noted as a limitation of the study. The specimens were subjected to conventional culture, biochemical testing, and ELISA at Dey Laboratory and the Microbiology Department of the Children’s Educational and Training Hospital in Tabriz, Iran.

Additionally, data including age, sex, clinical presentation, radiological findings, oxygen saturation levels, and treatment modalities were collected and recorded for all CF patients. Samples that tested positive via the molecular method were found to be positive either once or on multiple occasions.

#### Culture

Swabs were collected from the oral mucosa and transported to the Razi Vaccine and Serum Research Institute (RVSRI) reference laboratory. Samples were initially cultured in Brain Heart Infusion Broth (BHIB). After 24 h, a drop of the microbial suspension was subcultured onto non-selective media, including MacConkey agar (MAC) and nutrient agar supplemented with 5% sheep blood. The plates were incubated at 37 °C for 48 h. Phenotypic characterization and preliminary diagnostic tests, including Gram staining, were performed following incubation [7, 16].

Gram-negative bacilli and coccobacilli with a positive oxidase test were selected for biochemical identification. Tests included culturing on Triple Sugar Iron (TSI) agar, Kligler Iron Agar (KIA), and Simmons’ citrate agar; motility test; indole, Methyl Red (MR), Voges-Proskauer (VP); oxidation-fermentation (OF); arginine, ornithine, and lysine decarboxylation; and polymyxin resistance. All culture media and biochemical reagents were prepared in-house using commercial dehydrated powders (Merck, Germany) according to the manufacturer’s instructions and standard microbiological protocols [17]. Due to resource constraints, commercial ready-to-use culture media or automated identification systems could not be utilized; this limitation was noted in the study.

#### Molecular identification of BCC

##### Preparation of template DNA from conventional bacterial cultures

Template DNA was extracted from bacterial cultures according to standard protocols. The quantity and purity of the genomic DNA were assessed by measuring the absorbance at 260/280 nm [18].

##### PCR

The *23 S rDNA* gene of a wide range of *BCC* species was amplified via PCR using the CVMP 23 − 1 and CVMP 23-2b primer pair (Table 1). Positive isolates were further amplified using primers targeting the 5′- and 3′-ends of the *recA* gene locus, respectively (Table 2). PCR reactions were performed with a total volume of 50 µl. The reaction mixture contained 10 mM Tris-HCl (pH 8.3), 50 mM KCl, 200 µM of each dNTP, 150 nM of each primer, 1.5 mM MgCl2, 5% (v/v) DMSO, 2.5 U of *Taq* DNA polymerase, and 100 ng of purified DNA. Samples were initially denatured at 95 °C for 4 min. This was followed by 30 cycles of amplification with the following thermal profile: 30 s of denaturation at 95 °C, 30 s of annealing at 61 °C, and 30 s of extension at 72 °C. The PCR reaction was concluded with a final extension step at 72 °C for 10 min [18]. Due to budget constraints, sequencing to confirm the PCR-positive samples was not performed.

**Table 1:** Primers used for identification of the *23 S rDNA* gene in the *Burkholderia cepacia* complex *(BCC)* in people with Cystic Fibrosis (CF)

Primer	Target specices	Target	Primer sequences (5’→3’)	Amplicon size (bp)
*CVMP 23 − 1*	*BCC*	*23 S rRNA* (Intraspecies)	F: AAA CCG ACA CAG GTG G R: CAC CGA AAC TAG CA	526 (30)

**Table 2:** Primers used for species-specific identification of the *Burkholderia cepacia* complex *(BCC)* in people with Cystic Fibrosis (CF)

Primer	Target specices	Target	Primer sequences (5’→3’)	Amplicon size (bp)
*BKF*	*BCC*	*recA*	F: GGCNGAAGACGTCTACCGG R: TCGAAGTTGCTGCGCGAC	117 (30)

#### Design of an optimized indirect ELISA evaluation using native antigens for identification of the BCC

An optimized indirect ELISA using native antigens was developed to detect antibodies against *BCC*. Lipopolysaccharide (LPS) antigen was extracted from a standard *BCC* strain using the hot phenol–water method [19]. The extracted LPS was purified by dialysis and quantified using the Lowry method with bovine serum albumin (BSA) as the standard. Optimal antigen and antibody concentrations were determined by checkerboard titration [20]. For plate coating, microtiter wells were coated overnight at 4 °C with LPS diluted in carbonate–bicarbonate buffer (pH 9.6). After washing with PBS-T, the wells were blocked with 2% BSA for 1 h at room temperature. Serum samples diluted 1:50–1:200 in PBS-T containing 2% BSA were added and incubated for 30 min, followed by incubation with HRP-conjugated anti-human IgG (1:10,000). The reaction was developed with TMB substrate, stopped with 1 N H₂SO₄, and read at 450 nm using a microplate reader [19].

Cut-off values were established using receiver operating characteristic (ROC) curve analysis. Sensitivity and specificity were calculated from the ROC curve according to standard formulas. Cross-reactivity was evaluated against sera positive for *Burkholderia mallei* or *Burkholderia pseudomallei*, confirming high analytical specificity. Repeatability (intra- and inter-assay precision) was assessed using weakly, moderately, and strongly positive sera, with coefficients of variation (CV) below 15%, confirming high assay reliability.

## Results

Out of 100 oral mucosal swab samples, *BCC* was not isolated by MacConkey agar, blood agar, or via biochemical tests.

**Fig. 1:**
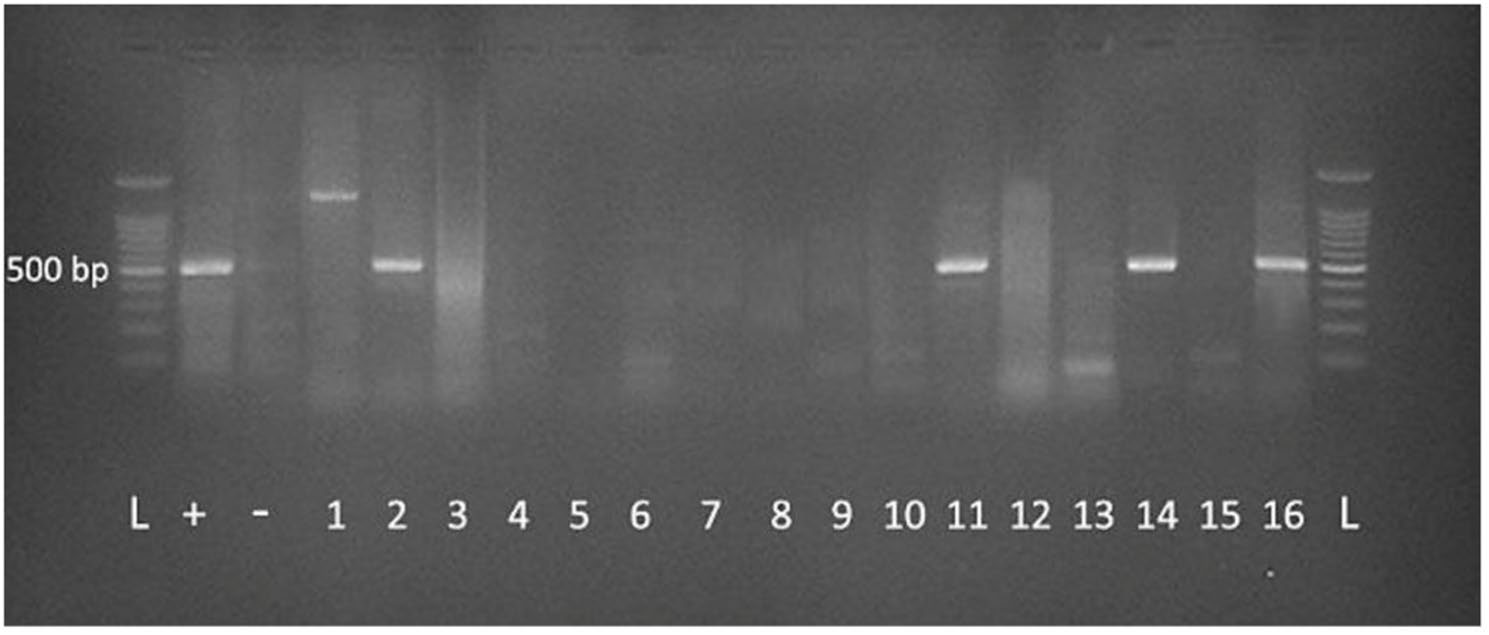
Agarose gel electrophoresis of PCR products from *Burkholderia cepacia* complex (*BCC*) isolates. Lane L: 100 bp DNA ladder (molecular weights are indicated in bp); Lane +: positive control (*BCC* DNA); Lanes 1–16: clinical isolates. The expected amplicon size of 350 bp is visible between the 400 and 300 bp markers

**Fig. 2:**
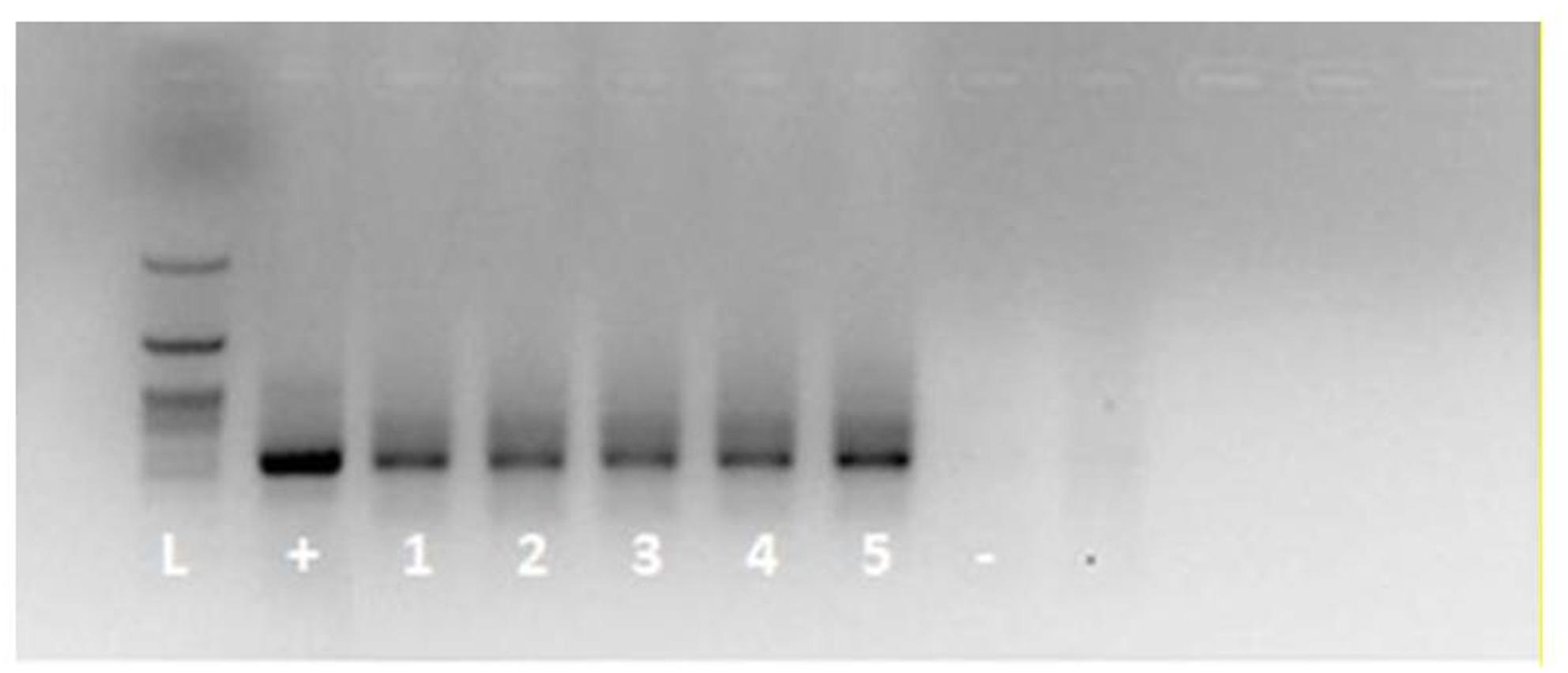
Agarose gel electrophoresis of PCR products from confirmed positive *Burkholderia cepacia* complex (*BCC*) isolates (selected from the isolates shown in Fig. 1). Lane L: 100 bp DNA ladder (molecular weights are indicated in bp); Lane +: Positive control (*BCC* DNA); Lanes 1–5: representative positive clinical isolates. The expected amplicon size was 350 bp, corresponding to the band located between the 400 and 300 bp markers

Following electrophoresis of the PCR products bands with a molecular weight of 350 bp (bp) were observed in five cases. Comparison with the positive *BCC* control confirmed the presence of *Burkholderia* DNA in these cases (Fig. 1). Representative electrophoresis results of the confirmed positive isolates are shown in Fig. 2.

Subsequently, the samples were examined using an indirect ELISA method, which identified 12 positive cases. This method demonstrated high sensitivity (92%) and specificity (90%) with an ROC-based cut-off of 0.90 and excellent reproducibility (CV < 15%). No cross-reactivity with *Burkholderia mallei* or *Burkholderia pseudomallei* was observed.

All five PCR-positive cases (aged 5–13 years) were also positive for ELISA; therefore, these cases were evaluated for clinical manifestations. Regarding clinical symptoms, growth delay, chronic cough, mucus production, and digital clubbing were reported. In terms of radiological findings, bronchiolar involvement, airway wall thickening, and bronchiectasis (observed via chest imaging) were noted in some cases, while oxygen saturation levels remained above 95%. All five patients with CF responded to co-trimoxazole treatment after three weeks, evidenced by improvements in clinical symptoms such as weight gain, improved appetite, and reduced cough and sputum production. All five patients demonstrated a favorable response to co-trimoxazole therapy (Table 3).

**Table 3:** Demographic data, clinical characteristics, radiological findings, and treatment outcomes of Cystic fibrosis patients with *Burkholderia cepacia* complex (*BCC*) infection

No.	Sex	Age (year)	Clinical presentation	*Chest CT-Scan results	+O2 sat (%)	¥Treatment	Outcome
1	Male	13	€Growth delay-mild digital clubbing- productive cough	Mild involvement in bronchioles- mild diffuse bronchiectasis	> 95	Co-trimoxazole tablets	£Recovery
2	Male	5	Growth delay- cough	Airway wall thickening- air trapping- without bronchiectasis	> 95	Co-trimoxazole tablets	Recovery
3	Female	8	Growth delay- clubbing- cough	Lung involvement in the form of thickening of bronchioles and airways- air trapping	> 95	Co-trimoxazole tablets	Recovery
4	Male	6	Growth delay	Without lung involvement	> 95	Co-trimoxazole tablets	Recovery
5	Male	10	Growth delay- cough	Airway wall thickening- air trapping- without bronchiectasis	> 95	Co-trimoxazole tablets	Recovery

*CT: computed tomography, +O2 sat: Oxygen saturation was measured by pulse oximetry at rest

€Growth delay: Defined as weight and height below the 5th percentile for age and sex according to standard growth charts., ¥Treatment: All patients received oral co-trimoxazole (Trimethoprim/Sulfamethoxazole) at a standard pediatric dosage of 8/40 mg/kg divided into two doses, administered for 14 days., £Recovery: Defined as complete resolution of clinical symptoms (cough, clubbing), or increasing of weight, and negative follow-up sputum cultures for *BCC* within 3 months post-treatment

Furthermore, the clinical data and treatment details for the five BCC-positive cases are presented in Table 3.

## Discussion

In this study, we failed to isolate *BCC* from people with CF using conventional culture methods; however, we obtained positive results using molecular analyses and ELISA. The failure to culture *BCC* was likely due to their fastidious growth requirements, the lack of selective culture media (e.g. BCSA), extensive antibiotic use among CF patients, the presence of viable but non-culturable (VBNC) forms, and potential issues with oral mucosal sample quality or delays in transport and processing. Therefore, the low identification rate should be interpreted with caution, as it may reflect methodological limitations rather than a true absence of *BCC*. In contrast, the positive PCR results reflect the high sensitivity of molecular methods for detecting bacterial DNA, even in the absence of viable cells [21].

Unlike *BCC*, which often establishes persistent and chronic colonization in the airways of people with CF, *P. aeruginosa* colonization can be intermittent and may not reach the high prevalence observed for *BCC* in some populations. This difference is clinically relevant because chronic *BCC* colonization is associated with more severe lung disease and a poorer prognosis in CF population [22].

The biochemical properties of *BCC* are inadequate for accurate identification and differentiation. Conventional tests cannot reliably diagnose this bacterium, even when using commercial systems [4, 5], due to a low bacterial load, previous or ongoing antibiotic therapy, and the use of non-selective or suboptimal media may have contributed to the failure to isolate *BCC* [23]. However, five *BCC* isolates were successfully identified using molecular method.

The variables examined in this study are essential for comparing efficient methods with serological assays optimized using native strains for ELISA kit development. By considering these findings, specialists can implement effective measures in epidemiological and diagnostic studies to ensure proper treatment. Furthermore, failure to accurately detect *BCC* may lead to an underestimation of infection prevalence, misclassification of patients’ risk status, and potentially inappropriate infection control measures. Although *BCC* is not a diagnostic criterion for CF itself, its presence significantly affects clinical management, treatment decisions, and patient prognosis, emphasizing the need for precise identification.

Vicenzi et al. collected 54 lung secretions samples from people with CF referred to a hospital [24]. Suspicious colonies were isolated after inoculating samples on the BCSA culture medium and incubating them. Bacterial DNA was extracted and the *recA* gene was examined by PCR. Of the 54 samples, 36 isolates were identified as *BCC* [24]. Other studies identified 4 and 12 isolates from 100 to 70 lung secretion samples from people with CF. In the present study, *BCC* was not isolated by culture or biochemical tests. However, five samples were identified as *BCC* by PCR [11, 25].

Eram et al.. isolated *BCC* from people with CF, confirming 5 of 53 samples. They suggested that BCSA medium can be used for the initial isolation and diagnosis of *BCC* [10].

Forouzeshfard et al. analyzed 27 throat swab mucosal samples from people with CF referred to Al-Zahra Hospital in Esfahan, Iran. *P. aeruginosa* was isolated from seven samples, while *BCC* was not isolated. Our results differ significantly from those reported by Forouzeshfard *et al.;* in our study, in addition to isolating *P. aeruginosa* in 40% of cases, *BCC* was also identified in 4% [26].

Da Silva Filho et al.. analyzed 106 mucosal swab and throat secretions from people with CF using PCR. Their results indicated that 56%, 4.3%, and 2.7% of samples were positive for *P. aeruginosa*, *BCC*, and *Stenotrophomonas*, respectively. Our results are consistent with those reported by Da Silva Filho et al.. regarding the isolation of *P. aeruginosa* and *BCC* from people with CF [27].

In this study, serological methods, including an optimized indirect ELISA using native antigens from reference strains, were compared with molecular detection techniques. Variables such as antigen purity, antibody dilutions, and assay threshold are critical for evaluating the efficiency, speed, and specificity of serological tests in detecting *BCC* infections.

These findings align with previous studies emphasizing the importance of purified antigens and proper cut-off determination for the serodiagnosis of *BCC* [5, 28, 29]. The discrepancies between PCR and ELISA results can be attributed to differences in their identification principles. ELISA reflects the host’s immune response to previous exposure or infection, whereas PCR detects the direct presence of bacterial genomic material. Cases that were ELISA-positive but PCR-negative may indicate past infection or bacterial clearance, while PCR-positive but ELISA-negative cases may correspond to the early stages of infection or patients with weakened immune systems [21].

## Conclusion

The present study demonstrated the diagnosis of *BCC* in patients with cystic fibrosis in northwestern Iran using serological and molecular methods, supported by clinical examinations and radiological findings.

In the current study, *BCC* was not identified using conventional methods; therefore, our inability to reliably isolate *BCC* likely reflects the lack of *BCC*-specific culture media, which was a limitation of this study. Molecular techniques such as PCR are recommended for accurate diagnosis, and in this study, *recA* gene analysis successfully identified *BCC*. Alongside molecular procedures, ELISA-based methods can identify *BCC* with high precision. These methods are suitable for diagnosis and yield easily interpretable results without the need for highly experienced staff or specialized equipment. Consequently, ELISA-based methods are efficient, rapid, specific, and cost-effective. Clinical evaluation by physicians is valuable for early diagnosis, allowing for targeted additional testing and the initiation of appropriate treatment, as well as for patient follow-up. It is recommended that future studies address the limitations of the current research, particularly regarding sampling methods and specific culture techniques in this age group, to allow for comparison with the present results.

### Limitations

This study has some limitations. Its cross-sectional design and small sample size, limited to a single referral center, make it difficult to generalize the findings to the broader CF population. The use of oral mucosal swabs instead of sputum or induced sputum was a limitation due to the young age of the patient and the difficulty of obtaining lower respiratory tract secretions that is associated with lower analytical sensitivity. Since *BCC* primarily colonizes the lower respiratory tract, oral mucosal sampling may have underestimated the true frequency of colonization. However, the successful detection of *BCC* in these samples underscores the potential value of this method for screening in young children unable to expectorate.

Additional technical limitations include the use of non-selective culture media lacking specific agents such as polymyxin, and a standardized incubation time that may have been insufficient for optimal recovery, as the study aimed to utilize standard conventional media available in the country. Due to budget constraints, the study relied on in-house prepared culture media and conventional biochemical reagents rather than commercial ready-to-use systems, and the absence of sequencing to confirm PCR-positive samples. Finally, the evaluation of ELISA using native strains was preliminary, and its diagnostic performance was not systematically compared with molecular methods.

## Data Availability

Data are available from the authors upon reasonable request.

## Abbreviations

PCR: Polymerase Chain Reaction
ELISA: Enzyme-Linked Immunosorbent Assay
CF: Cystic fibrosis
BCC: *Burkholderia cepacia* complex
BC: *Burkholderia cepacia*
CFTR: Cystic fibrosis transmembrane conductance regulator
P. aeruginosa: *Pseudomonas aeruginosa *
CT scan: Computed tomography scan
RVSRI: Razi Vaccine and Serum Research Institute
BHIB: Brain Heart Infusion Broth
MAC: MacConkey agar
TSI: Triple Sugar Iron
KIA: Kligler Iron Agar
MR test: Methyl Red test
VP test: Voges-Proskauer test
